# SPARCL1 Is a Novel Prognostic Biomarker and Correlates with Tumor Microenvironment in Colorectal Cancer

**DOI:** 10.1155/2022/1398268

**Published:** 2022-01-24

**Authors:** Hai-Ping Zhang, Jun Wu, Zhi-Feng Liu, Jing-Wen Gao, Shu-Yu Li

**Affiliations:** Department of Gastroenterology, Hubei No. 3 People's Hospital of Jianghan University, Wuhan, China

## Abstract

**Background:**

Secreted protein acidic and rich in cysteine-like 1 (SPARCL1) plays an important role in tumor pathogenesis. We aim to evaluate the clinical significance and potential biological roles of SPARCL1 in colorectal cancer (CRC).

**Methods:**

Datasets from the Cancer Genome Atlas (TCGA) and Gene Expression Omnibus (GEO) databases were downloaded to evaluate the expression levels of SPARCL1 in CRC. Receiver operating characteristic (ROC) curve was constructed to evaluate the diagnostic value of SPARCL1. Then, comprehensive database search was conducted for published clinical studies to explore clinical significance of SPARCL1. In addition, coexpression genes of SPARCL1 were identified through the cBioPortal database and enrichment analysis of SPARCL1 and its coexpression genes were performed by the “clusterProfiler” R package. Finally, the correlations between SPARCL1 and tumor microenvironment scores, tumor-infiltrating immune cells in CRC were determined by “ESTIMATE” and “GSVA” R packages.

**Results:**

SPARCL1 was significantly downregulated in CRC tissues, and SPARCL1 showed high accuracy for diagnosis of primary CRC in both GEO and TCGA datasets. Pooled results from published clinical studies showed SPARCL1 expression was associated with differentiation (OR = 1.89, 95% CI: 1.38-2.59), tumor stage (OR = 0.47, 95% CI: 0.29-0.77), distant metastasis (OR = 0.53, 95% CI: 0.33-0.84), and overall survival (HR = 0.56, 95% CI: 0.43-0.74). SPARCL1 and its top 300 coexpression genes were involved in several KEGG pathways, such as focal adhesion, cell adhesion molecules, PI3K-Akt signaling pathway, cGMP-PKG signaling pathway, and ECM-receptor interaction. Besides, the SPARCL1 expression was significantly correlated with stromal score, immune score, ESTIMATE score, and diverse immune cells.

**Conclusion:**

SPARCL1 significantly correlated with clinicopathological features and tumor microenvironment in CRC.

## 1. Introduction

Colorectal cancer (CRC) is the third most common cancer worldwide, ranking as high as the second leading cause of cancer-related deaths worldwide [1, 2]. Despite a decrease in CRC incidence during the last decades [2–4], the disease was still associated with unacceptably high morbidity and mortality, which brought great burden to global health and economy [4–6]. About 26.5% of CRC patients were diagnosed with liver metastases, and the 5-year survival of patients with liver metastases treated with resection was 48.6% [7]. Therefore, identification of the molecular mechanism during CRC pathogenesis, as well as identification of potential prognostic biomarkers, is still needed.

Secreted protein acidic and rich in cysteine-like 1 (SPARCL1) is one of the members of the SPARC-related family, which play an important role in the regulation of cell adhesion, migration, and proliferation [8]. Up to now, SPARCL1 has been reported to be downregulated in several human malignancies and correlated with poor prognosis [9]. Limited studies evaluated the relationship between SPARCL1 expression and CRC clinicopathological factors, and the results were not always consistent [10–13]. Some studies showed that SPARCL1 lower expression indicated poor prognosis [10, 11, 13], but some results did not show this significance [12]. Therefore, more studies are needed to evaluate the expression and prognostic role of SPARCL1 in CRC.

Tumor environment consists of epithelial cancer cells, stromal fibroblasts, and multiple immune cells, which could predict the cancer prognosis and therapeutic [14, 15]. In recent years, immune checkpoint inhibitors show great potential for the treatment of various cancers. Targets for immune checkpoint blockade therapy, such as PDCD1 and CD274, have been approved for the treatment of advanced CRC [16]. One study showed that SPARCL1 could regulate tumor microenvironment-dependent endothelial cell heterogeneity in CRC [17]. In the present study, we aim to evaluate the expressions and prognostic roles of SPARCL1 in CRC patients through integrated bioinformatic analysis and clinical studies. Additional efforts were made to explore the potential biological role and the correlation with tumor microenvironment.

## 2. Materials and Methods

### 2.1. SPARCL1 Expression Analysis

Level 3 RNA sequencing data of CRC patients from TCGA were downloaded through UCSC Xena (https://xenabrowser.net/) [18]. Expression values were transformed into Log2 transcripts per kilobase million value. Gene expression of GEO datasets was downloaded from GEO database (https://www.ncbi.nlm.nih.gov/geo/) [19], including GSE9348, GSE23878, GSE24514, GSE49355, and GSE81558. The probe annotation in GEO datasets was performed according to each platform. Log2 transformation was applied for normalization of gene expression values in all the GEO datasets. The expression levels of SPARCL1 between CRC tissues and normal tissues were compared by *t*-test. Then, receiver operating characteristic (ROC) curves were constructed to evaluate the diagnostic value of SPARCL1 for primary CRC. The area under the ROC curve (AUC) and *p* values were calculated.

### 2.2. Literature Search of Clinical Significance of SPARCL1 in CRC

A comprehensive search of PubMed and Wanfang databases was performed to include the studies evaluating the expression and prognosis of SPARCL1 in CRC. The keywords were listed as the following: “colon cancer”, “rectal cancer”, “colon adenocarcinoma”, “rectal adenocarcinoma”, “colorectal cancer”, “colorectal adenocarcinoma”, “SPARCL1”, “expression”, “prognosis”, “clinical outcome”. Pooled analysis was performed by STATA version 14.0 software (Stata Corporation, College Station, Texas, USA). Pooled hazard ratio (HR) was calculated to evaluate the prognostic role of SPARCL1 in CRC. Pooled odds ratio (OR) was calculated to determine the correlation of SPARCL1 expression with distant metastasis, lymph node metastasis, tumor differentiation and tumor stage. Heterogeneity across studies was assessed using the Chi-square-based *Q* statistical test. A random-effects model was used when heterogeneity was present (*p* < 0.05 and/or *I*^2^ > 50%); otherwise, a fixed-effects model (Mantel-Haenszel method) was applied.

### 2.3. Coexpression and Enrichment Analysis

Coexpression genes of SPARCL1 were identified through the cBioPortal database (http://www.cbioportal.org) [20]. Coexpression genes of SPARCL1 were selected according to Spearman correlation coefficient and adjusted p value. Gene Ontology (GO) and Kyoto Encyclopedia of Genes and Genomes (KEGG) enrichment analyses were performed to explore the potential functions and pathways of the coexpression genes. GO terms were divided into three classifications: biological process (BP) term, molecular function (MF) term, and cellular component (CC) term. The enrichment analyses were performed via the “clusterProfiler” R package [21]. Adjusted p < 0.05 is considered as significant enrichment term.

### 2.4. Correlation of SPARCL1 with Tumor Microenvironment

Tumor microenvironment status could influence tumor progression and therapy response [15, 16]. The correlation of SPARCL1 with tumor environment was further explored. Tumor microenvironment scores, including stromal score, immune score, and ESTIMATE score, were evaluated by the “ESTIMATE” R package [22]. Immune cells are important components in tumor microenvironment. The infiltration levels of immune cells were estimated by the ssGSEA method through the “GSVA” R package [23]. The gene markers of immune cells were referenced in a prior study [24]. Immune checkpoint genes were biomarkers of immunotherapy [25]. The correlations of SPARCL1 expression with tumor microenvironment scores, infiltration levels of immune cells, and the expression levels of immune checkpoint genes were evaluated by the Spearman correlation test.

## 3. Results

### 3.1. Expression of SPARCL1 in CRC

**Figure 1:**
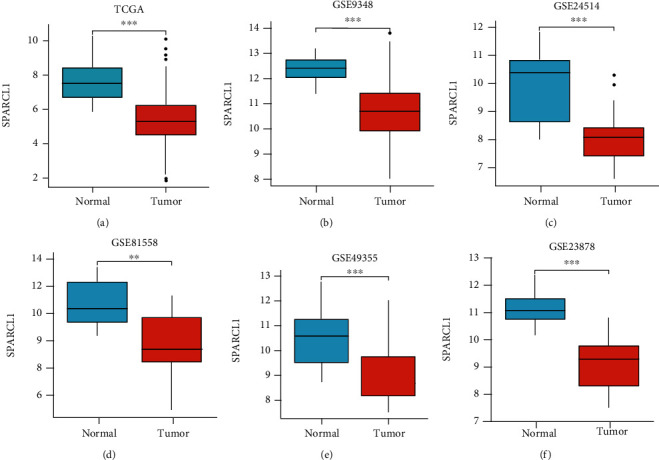
SPARCL1 expression in different datasets. (a–f) SPARCL1 expression between CRC and normal tissues in TCGA, GSE9348, GSE24514, GSE81558, GSE49355, and GSE23878 datasets. ^∗^*P* < 0.05, ^∗∗^*P* < 0.01, and ^∗∗∗^*P* < 0.001.

**Figure 2:**
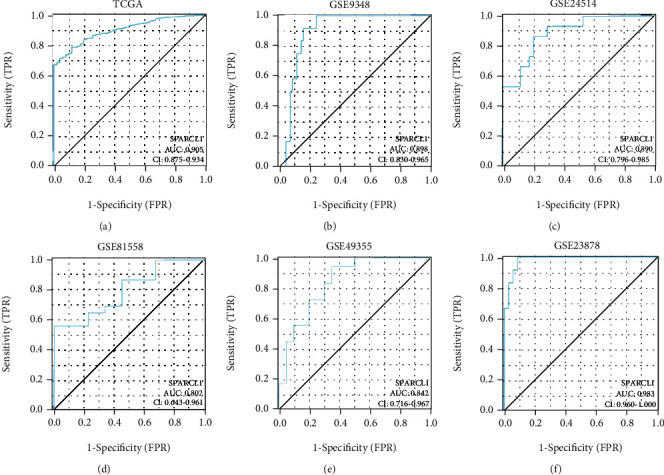
ROC curves of SPARCL1 for CRC patients. (a–f) Diagnosis value of SPARCL1 for primary CRC in TCGA, GSE9348, GSE24514, GSE81558, GSE49355, and GSE23878 datasets.

**Figure 3:**
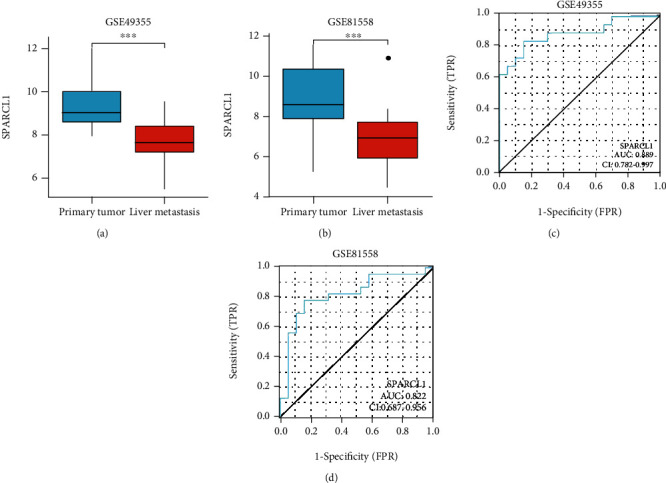
SPARCL1 expression between primary tumor and liver metastasis tissues. (a, b) SPARCL1 was downregulated in liver metastasis tissues in GSE49355 and GSE81558 datasets; (c, d) diagnostic value of SPARCL1 for liver metastasis in GSE49355 and GSE81558 datasets. ^∗^*P* < 0.05, ^∗∗^*P* < 0.01, and ^∗∗∗^*P* < 0.001.

The expressions of SPARCL1 between primary CRC and normal colorectum tissues were analyzed in TCGA, GSE9348, GSE23878, GSE24514, GSE49355, and GSE81558 datasets. SPARCL1 expression was significantly lower in primary CRC tissues than those in normal colorectum tissues (Figures 1(a)–1(f)). Furthermore, SPARCL1 showed high accuracy for diagnosis of primary CRC in the above datasets (Figures 2(a)–2(f)). We also found that SPARCL1 was downregulated in CRC liver metastasis tissues and had accuracy for diagnosis of CRC liver metastasis (Figures 3(a)–3(d)). These results showed that SPACRL1 was a reliable diagnosis biomarker for CRC.

### 3.2. Validation in Clinical Studies

**Figure 4:**
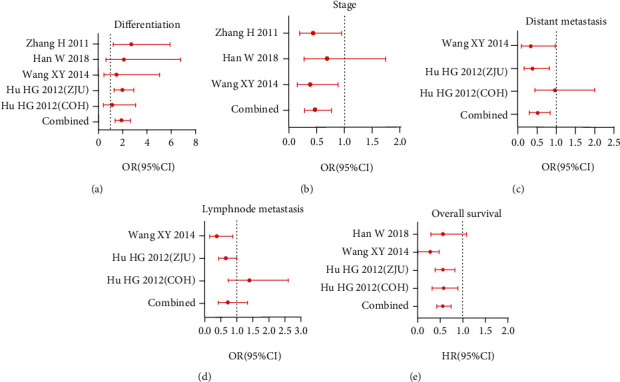
Relationship between SPARCL1 and clinicopathological features in CRC patients in published clinical studies. The correlation of SPARCL1 expression with (a) tumor differentiation, (b) tumor stage, (c) distant metastasis, (d) lymph node metastasis, and (e) overall survival.

Four studies [10–13], containing five datasets, were included for further analysis. SPARCL1 expression was estimated by immunohistochemistry scores in four studies. The clinical significance of SPARCL1 in individual study is described in Table 1. SPARCL1 expression was related to distant metastasis and lymph node metastasis in two studies [10, 11], duke stage in two studies [11, 13], and differentiation in two studies [10, 13]. Pooled results showed SPARCL1 expression was associated with differentiation (OR = 1.89, 95% CI: 1.38-2.59, *p* = 0.001), tumor stage (OR = 0.47, 95% CI: 0.29-0.77, *p* = 0.002), and distant metastasis (OR = 0.53, 95% CI: 0.33-0.84, *p* = 0.007) (Figures 4(a)–4(c)). However, SPARCL1 expression was not related to lymph node metastasis (OR = 0.73, 95% CI: 0.39-1.37, *p* = 0.329) (Figure 4(d)). SPARCL1 low expression predicted poor survival (HR = 0.56, 95% CI: 0.43-0.74, *p* ≤ 0.01) (Figure 4(e)). Therefore, SPARCL1 might be a potential prognostic biomarker for CRC patients.

### 3.3. Biological Roles of SPARCL1

**Figure 5:**
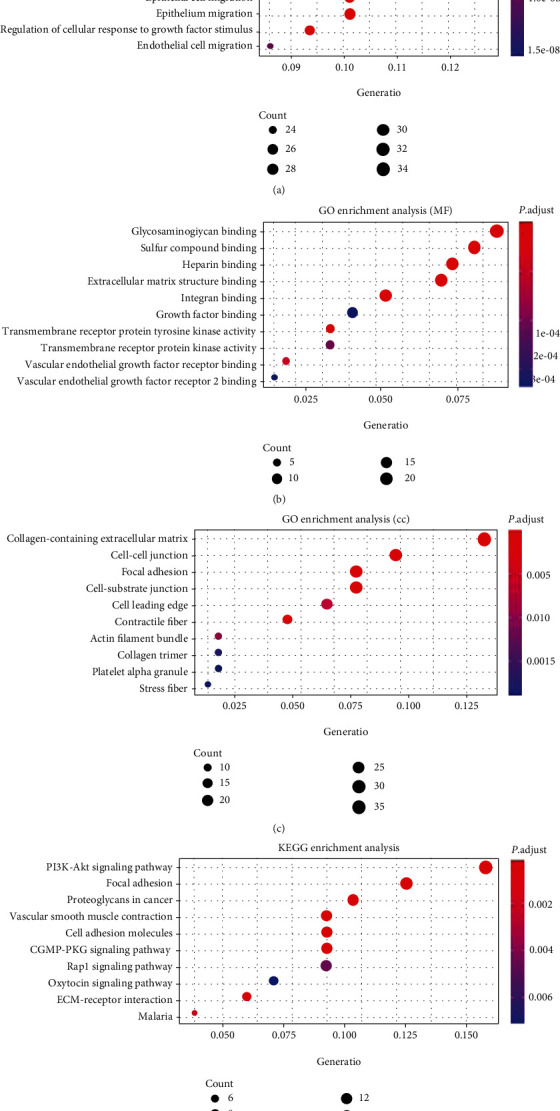
Enrichment analysis of SPARCL1 and its coexpression genes: (a) biological process; (b) molecular function; (c) cellular component; (d) KEGG pathways.

The top 300 coexpression genes of SPARCL1 were identified through the cBioPortal database. The top 300 coexpression genes and SPARCL1 were employed for further enrichment analysis. Among all the significant enrichment analysis terms, those genes were mainly enriched in extracellular matrix regulation and cell adhesion. Top 10 MF terms, BP terms, CC terms, and KEGG pathways are described in Figures 5(a)–5(d). Extracellular matrix organization, glycosaminoglycan binding, and collagen-containing extracellular matrix were the most significant BP term, MF term, and CC terms, respectively. Several KEGG pathways were involved, such as focal adhesion, cell adhesion molecules, PI3K-Akt signaling pathway, cGMP-PKG signaling pathway, and ECM-receptor interaction.

### 3.4. Correlation of SPARCL1 with Tumor Microenvironment

**Figure 6:**
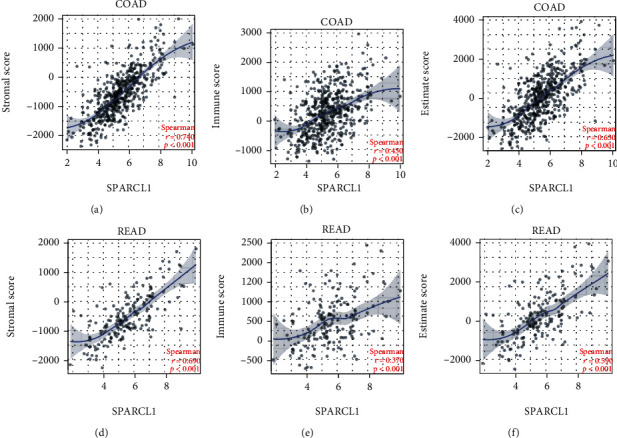
Correlation of SPARCL1 with tumor microenvironment scores: (a–c) correlation of SPARCL1 with stromal score, immune score, and ESTIMATE score in COAD; (d–f) correlation of SPARCL1 with stromal score, immune score, and ESTIMATE score in READ.

**Figure 7:**
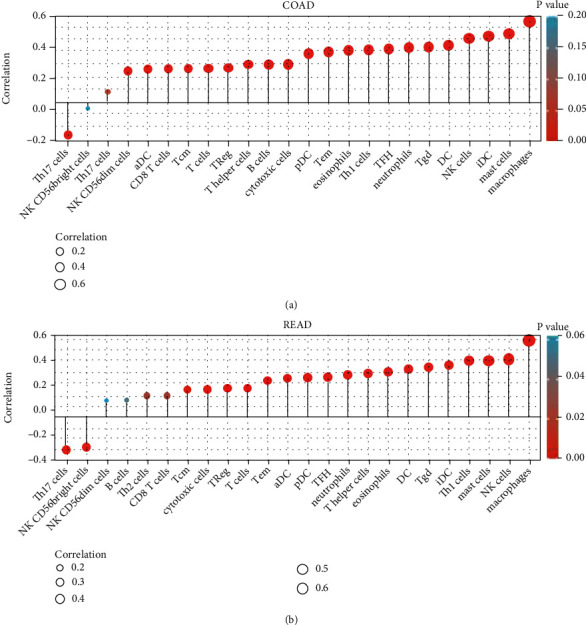
Correlation of SPARCL1 with immune cells: (a) correlation of SPARCL1 with immune cells in COAD; (b) correlation of SPARCL1 with immune cells in READ.

The tumor microenvironment scores of CRC samples were estimated by the “ESTIMATE” R package. SPARCL1 was correlated with stromal score, immune score, and ESTIMATE score in both colon adenocarcinoma (COAD) and rectum adenocarcinoma (READ) (Figure 6). The infiltration levels of immune cells in tumor microenvironment were further estimated. To make reliable immune infiltration estimations, we utilized the ssGSEA method through the “GSVA” R package to estimate the infiltration levels of different immune cells. SPARCL1 was significantly correlated with the B cells, CD4+ T cells, CD8+ T cells, macrophages, neutrophils, NK cells, and dendritic cells in both COAD and READ (Figure 7). Among the various immune cells, SPARCL1 showed strong correlations with macrophages. The above results indicated that SPARCL1 might play important roles in tumor microenvironment.

**Figure 8:**
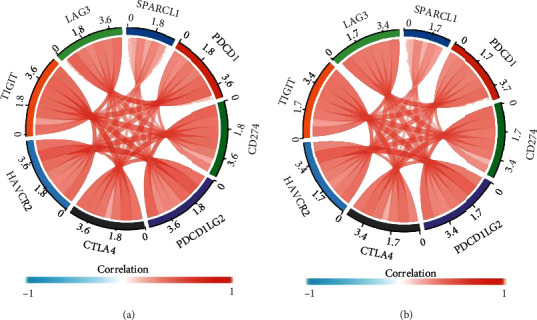
Correlation of SPARCL1 with immune checkpoints: (a) correlation of SPARCL1 with immune checkpoints in COAD; (b) correlation of SPARCL1with immune checkpoints in READ.

In order to further explore the value of SPARCL1 for immune target therapy, we explored the correlations of SPARCL1 with main immune checkpoints, including PDCD1, CD274, PDCD1LG2, CTLA4, HAVCR2, TIGIT, and LAG3. SPARCL1 positively correlated with the expression of the above immune checkpoints in both COAD and READ (Figure 8).

## 4. Discussion

In the present study, we evaluated the clinical significance and biological roles of SPARCL1 through integrated bioinformatic analysis and pooled clinical studies. Our studies demonstrated that SPARCL1 significantly correlated with clinicopathological features, overall survival, and tumor microenvironment in CRC, which might help us better understanding the roles of SPARCL1 in CRC.

Alteration of SPARCL1 has been found to be involved in malignancies. Decreased SPARCL1 expression has been reported in gastric cancer [26, 27], liver cancer [28], cholangiocarcinoma [29], pancreatic cancer [30], breast cancer [31], and prostate cancer [32]. In our study, five GEO datasets and one TCGA dataset were included to evaluate the expressions of SPARCL1 in CRC. SPARCL1 low expression was detected in primary CRC vs. normal colorectum and CRC liver metastasis vs. primary CRC. Besides, ROC analyses demonstrated that SPARCL1 had high accuracy for primary CRC and CRC liver metastasis. The above results indicated that SPARCL1 might be a potential diagnostic marker for primary CRC and CRC with liver metastasis.

To date, limited studies have reported that lower SPARCL1 expression suggests poor survival in human malignancies [27–32]. A meta-analysis revealed that SPARCL1 could predict poor clinical outcomes for gastrointestinal malignancies [33]. In our study, we pooled four clinical studies to explore the prognostic value of SPARCL1 in CRC. Regarding our results, we found that the SPARCL1 expression was associated with distant metastasis, tumor differentiation, tumor stage, and overall survival, which shared similar results with the previous study [33]. Therefore, the SPARCL1 expression might be employed as a potential prognosis marker for patients with CRC.

However, the mechanism of SPARCL1 in human cancers remains unclear. In order to explore the potential biological roles of SPARCL1, we analyzed the coexpression genes of SPARCL1 and enrichment analysis was performed. SPARCL1 and its top 300 coexpression genes were significantly enriched in extracellular matrix regulation and cell adhesion, which was in consistent with previous studies [8]. SPARCL1 is considered to be a potential tumor suppressor gene and participates in tumor development, by regulating tumor cell growth and proliferation [34]. Furthermore, SPARCL1 negatively regulated tumor cell migration and invasiveness in vitro and tumor metastatic growth in vivo [35, 36]. A study showed that SPARCL1 could inhibit tumor growth and liver metastasis in a mouse xenograft model and induce differentiation through mesenchymal-epithelial transition in colon cancer cells [10]. The above biological behaviors might explain the relationship between SPARCL1 and clinicopathological features of CRC patients. Besides, SPARCL1 may be involved in the regulation of drug resistance in cancer. One study showed that SPARCL1 was involved in the regulation of drug resistance in ovarian cancer by comprehensive bioinformatic analysis [37].

Considering the importance of tumor environment during cancer pathogenesis, the roles of SPARCL1 in tumor environment were comprehensively evaluated. Our study demonstrated that SPARCL1 correlated with stromal score, immune score, and ESTIMATE score, which indicated that SPARCL1 might play important roles in tumor environment. One study showed that SPARCL1 could regulate tumor microenvironment-dependent endothelial cell heterogeneity in CRC [17], making our results more convincing. Immune cell infiltration of CRC is closely associated with clinical outcome [38, 39]. We further explored the correlations between SPARCL1 and tumor-infiltrating immune cells in CRC. Our study showed that SPARCL1 expression was significantly related to the infiltration level of B cells, CD4+ T cells, CD8+ T cells, macrophages, neutrophils, NK cells, and dendritic cells in CRC. SPARCL1 had the strongest correlation with macrophages in both COAD and READ. One recent study demonstrated that tumors lacking M1 macrophages or with an increased number of M2 macrophages, eosinophils, and neutrophils were associated with the poor prognosis [38]. Another study showed that neutrophils and macrophages were significantly correlated with prognosis in CRC [39]. Furthermore, SPARCL1 positively correlated with the expression of immune checkpoint genes, including PDCD1, CTLA4, LAG3, and HAVCR2. The above results might indicate that SPARCL1 is a potential tumor immune therapy target.

## 5. Conclusion

SPARCL1 is downregulated in CRC patients and could provide high accuracy for diagnosis of primary CRC and CRC with liver metastasis. Furthermore, SPARCL1 downregulation is significantly associated with poor prognosis. Besides, SPARCL1 is correlated with tumor environment in CRC. The information we have obtained might shed light on clinical application and future research. Nevertheless, more experimental studies are needed to further validate these findings.

## Figures and Tables

**Table 1:** The correlation between SPARCL1 protein expression and clinicopathological features.

		Total	High (positive) expression	Low (negative) expression	*p* value
Hu HG 2012(COH) (10)	Distant metastasis				0.911
	Yes	42	12	30	
	No	180	53	127	

Hu HG 2012(ZJU) (10)	Distant metastasis				0.01
	Yes	40	10	30	
	No	372	172	200	

Wang XY 2014 (11)	Distant metastasis				0.039
	Yes	28	5	23	
	No	76	30	46	

Hu HG 2012(COH) (10)	Lymph node metastasis				0.322
	Yes	55	19	36	
	No	167	46	121	

Hu HG 2012(ZJU) (10)	Lymph node metastasis				0.049
	Yes	183	71	112	
	No	229	111	118	

Wang XY 2014 (11)	Lymph node metastasis				0.022
	Yes	69	18	51	
	No	35	17	18	

Han W 2018 (12)	Duke stage				0.421
	A + B	44	19	25	
	C + D	35	12	23	

Zhang H 2011 (13)	Duke stage				0.032
	A + B	87	75	12	
	C + D	77	56	21	

Wang XY 2014 (11)	Duke stage				0.022
	A + B	35	17	18	
	C + D	69	18	51	

Hu HG 2012(COH) (10)	Differentiation				0.852
	High	19	6	13	
	Low	193	57	136	

Hu HG 2012(ZJU) (10)	Differentiation				0.001
	High	159	86	73	
	Low	253	96	157	

Wang XY 2014 (11)	Differentiation				0.536
	High	12	5	7	
	Low	91	30	61	

Han W 2018 (12)	Differentiation				0.238
	High	13	7	6	
	Low	66	24	42	

Zhang H 2011 (13)	Differentiation				0.012
	High	114	97	17	
	Low	50	34	16	

## Data Availability

The SPARCL1 expression data were obtained through UCSC Xena (https://xenabrowser.net/) and GEO database (https://www.ncbi.nlm.nih.gov/geo/). The coexpression genes of SPARCL1 were assessed using the cBioPortal database (http://www.cbioportal.org). Four clinical studies, which evaluated the clinical significance of SPARCL1 in CRC, were downloaded from PubMed (https://pubmed.ncbi.nlm.nih.gov/). The data of the correlation between SPARCL1 expression and distant metastasis, lymph node metastasis, or tumor differentiation and tumor stage were obtained from the four clinical studies.
